# Anti-Inflammatory, Immunomodulatory, and Antioxidant Activities of Allicin, Norfloxacin, or Their Combination against *Pasteurella multocida* Infection in Male New Zealand Rabbits

**DOI:** 10.1155/2018/1780956

**Published:** 2018-06-27

**Authors:** Rasha T. M. Alam, Elshaima M. Fawzi, Maha I. Alkhalf, Wafa S. Alansari, Lotfi Aleya, Mohamed M. Abdel-Daim

**Affiliations:** ^1^Department of Clinical Pathology, Faculty of Veterinary Medicine, Zagazig University, Zagazig, 44511 Sharkia, Egypt; ^2^Department of Internal Medicine (Infectious Diseases), Faculty of Veterinary Medicine, Zagazig University, Zagazig, 44511 Sharkia, Egypt; ^3^Biochemistry Department, Faculty of Science-Al Faisaliah, King Abdulaziz University, Jeddah, Saudi Arabia; ^4^Bourgogne Franche-Comté University, Chrono-Environnement Laboratory, UMR CNRS 6249, 25030 Besançon Cedex, France; ^5^Department of Pharmacology, Faculty of Veterinary Medicine, Suez Canal University, Ismailia 41522, Egypt

## Abstract

The present study investigated the efficacy of allicin as an antibacterial, anti-inflammatory, antioxidant, and immunostimulant agent in reducing the severity of *Pasteurella multocida* (*P. multocida*) type B infection in rabbits. Fifty New Zealand rabbits, 5 weeks old, were divided equally into five groups. Except for group 1, all groups were intranasally infected with *P. multocida* type B (2 × 10^5^ colony forming units/ml/rabbit). Then, group 3 rabbits were orally treated with allicin (50 mg/kg BW) for 5 days, group 4 rabbits received a single oral dose of norfloxacin 30% (100 mg/kg BW), while group 5 rabbits were treated with a combination of norfloxacin and allicin. Hematological, serum biochemical, inflammatory cytokine, immunological, and histopathological analyses were performed. Results revealed that rabbits, infected with *P. multocida* type B, exhibited macrocytic hypochromic anemia and leukocytosis with a significant elevation in the phagocytic percentage and index. Moreover, significant reductions in serum total protein, albumin, globulin, and immunoglobulin (IgG and IgM) levels were observed in infected rabbits. Infected rabbits showed significant increases in serum inflammatory cytokine (TNF-*α* and IL-6), alanine aminotransferase, alkaline phosphatase, lactate dehydrogenase, and serum bilirubin (total, direct, and indirect) levels. Further, *P. multocida* infection induced oxidative stress as demonstrated by the significant reduction in serum levels of reduced glutathione and superoxide dismutase enzyme and marked elevation in serum malondialdehyde. Treatment with allicin, norfloxacin, or their combination significantly ameliorated the alterations in all studied parameters. In conclusion, allicin could ameliorate the inflammation and oxidative stress, induced by *P. multocida* type B infection in rabbits.

## 1. Introduction

*Pasteurella multocida (P. multocida)* is a microbe, which occurs naturally in the respiratory tract of some animal species; however, it can be a virulent pathogen that infects other animal hosts [1]. Pasteurellosis is a disease that affects rabbits, characterized by rhinitis, pneumonia, orchitis, otitis media, septicemia, and abscess formation [2]; however, infection with *P. multocida* may be asymptomatic [3].

Norfloxacin is a second-generation fluoroquinolone with a broad spectrum of activity, potent bactericidal action, and high tissue penetration [4, 5]. Despite its efficacy, it has been shown that antibiotic traces which are present in animal products can affect human health directly due to side effects and impact on intestinal flora and precipitation or indirectly through increasing bacterial resistance to antibiotics (maximum residue limit for norfloxacin is 0.02–0.1 in pig and poultry) [6]. The emergence of antibiotic resistance [7] and the rabbits' hypersensitivity to several antimicrobial agents precludes the extensive applications of antibiotics in this species [8].

Garlic (*Allium sativum*) is considered the oldest medicinal herb and had been used for the treatment of several diseases. It is effective against several gram-positive, gram-negative, and acid-fast bacteria [9]. Moreover, it has antioxidant, immunomodulatory, and anti-inflammatory effects [10–12]. Allicin is a major biologically active component of garlic clove extracts with a potent antioxidant activity [11]. Moreover, it has been shown to exert organoprotective effects against several xenobiotics, such as deltamethrin and doxorubicin [13, 14].

This study was performed to evaluate the ameliorative (antioxidant, anti-inflammatory, and immunomodulatory) effects of allicin alone or combined with norfloxacin in the treatment of *P. multocida* infection in rabbits.

## 2. Material and Methods

### 2.1. Experimental Animals

Male New Zealand white rabbits (850–1000 g, 5 weeks of age) were obtained from the Faculty of Veterinary Medicine, Zagazig University. Rabbits were housed in a pathogen-free facility, maintained at 24 ± 2°C with a 50–60% relative humidity and a 12 h light: dark cycle. Rabbits had ad libitum access to tap water and basal ration. All rabbits were acclimatized for one week before beginning the experiment. Rabbit handling and treatment procedures were performed according to the Guidelines for the Care and Use of Laboratory Animals of the National Institutes of Health (NIH) and approved by a research ethics committee at the Faculty of Veterinary Medicine, Zagazig University. All efforts were exerted to reduce animal suffering.

### 2.2. Bacterial Strain

*Pasteurella multocida type B* was obtained from the National Research Centre, Dokki (Giza, Egypt) and used for experimental infection with a final concentration of 2 × 10^5^ colony-forming units (CFU) prior to inoculation.

### 2.3. Drugs

Norfloxacin was purchased from ATCO Pharma (Atonor 30, 300 mg/ml, oral suspension). Allicin was obtained from Anhui Ruisen Biological Technology Co., China. All used kits were marketed by Spinreact, Spain.

### 2.4. Experimental Design

After acclimatization, fifty rabbits (6 weeks of age) were randomly allocated into five equal groups (10 rabbits for each group). Group 1 rabbits were given intranasal (IN) phosphate-buffered saline (1 ml) and kept as a negative control group. Rabbits in other groups were IN infected with 2 × 10^5^ CFU/ml/rabbit of *P. multocida type B* at day 1 of the experiment [15]. Five days later (after appearance of clinical signs), group 3 rabbits were orally treated with allicin 50 mg/kg body weight for 5 days [16], group 4 rabbits received a single oral dose of norfloxacin (100 mg/kg body weight) [17], while group 5 rabbits were orally treated with a combination of norfloxacin and allicin in the same dose regimen used for groups 3 and 4.

### 2.5. Sample Collection and Preparation

Blood samples were collected at the end of the experiment (24 hours after last dose) from the ear vein: 2 ml of blood in a heparinized test tube for evaluation of phagocytic percent and index, 0.5 ml in a test tube with the anticoagulant (EDTA) for hematological studies, and 5 ml in a glass tube without anticoagulant for serum separation to assess biochemical parameters. The lung and liver tissues were dissected out after cervical dislocation, washed with physiological saline, and fixed with 10% neutral buffered formalin for histopathological examination.

### 2.6. Phagocytosis Assay

The heparinized blood samples of rabbits from different groups were used for leukocyte separation. *Candida albicans (C. albicans)* was prepared and used for evaluation of the phagocytic activity by the method of Wilkinson [18]. The number of macrophages (neutrophils and/or monocytes) containing *C. albicans* (phagocytic%) that attached to or were ingested by 100 phagocytes in each individual preparation was determined by light microscopy. Moreover, the phagocytic index was calculated by determining the average number of attached and engulfed *C. albicans* multiplied by the phagocytic percent [19].

### 2.7. Hematological Indices

Blood samples, collected in tubes containing 10% EDTA solution, were used for determination of red blood cells (RBCs), hemoglobin (Hb), hematocrit/packed cell volume (HCT/PCV%), white blood cells (WBC: leukogram), and differential leukocyte counts (DLC) according to Coles [20]. The mean corpuscle volume (MCV) and mean corpuscle hemoglobin concentration (MCHC) were calculated.

### 2.8. Biochemical Assay

Serum samples were analyzed for determination of alanine aminotransferase (ALT) by the method of Reitman and Frankel [21], alkaline phosphatase (ALP) according to Tietz [22], and lactate dehydrogenase (LDH) enzyme according to Buhl and Jackson [23]. The total protein and albumin levels were measured according to Doumas et al. [24] and Drupt [25], respectively. The globulin concentration was calculated by subtracting the obtained albumin concentration from the total protein concentration according to Doumas and Biggs [26].

### 2.9. Immunoglobulin and Cytokine Assay

The serum concentrations of immunoglobulin G (IgG) and immunoglobulin M (IgM) were determined using the commercial IgG and IgM ELISA kits, purchased from Bethyl Laboratories, USA (cat. no. E121-111, lot no. E121-111-150331, and cat. no. E120-110, lot no. E120-110-29), respectively. The serum concentrations of tumor necrosis factor-alpha (TNF-*α*) and interleukin-6 (IL-6) were measured using standard kits obtained from Genorise (Nori™ Rabbit TNF-*α* ELISA Kit, cat. no. GRC144010) and CUSABIO (CSB-E06903Rb), respectively.

### 2.10. Estimation of Serum Oxidative Stress Markers

Serum samples were used to assay the reduced glutathione (GSH) level according to Jollow et al. [27], superoxide dismutase (SOD) activity according to Nishikimi et al. [28], and the lipid peroxidation marker (malondialdehyde (MDA)) depending on the thiobarbituric acid reactivity using the method of Ohkhawa et al. [29].

### 2.11. Histopathological Investigation

Specimens from the lungs and liver of different groups were collected and fixed in 10% neutral buffered formalin, transferred in ethanol (70%), then cleared in xylene and embedded in paraffin. Five-micron-thick sections of paraffin were prepared then stained with hematoxylin and eosin dyes [30] and examined microscopically.

### 2.12. Statistical Analysis

All data were expressed as the mean ± standard error of mean (SEM) and were statistically analyzed by the SPSS/PC software (2001) using one-way analysis of variance (ANOVA), followed by post hoc Tukey's test. A *p* value < 0.05 was considered statistically significant.

## 3. Results

### 3.1. Clinical Signs and Mortality Rates

On the third day of the experiment, gp.2 rabbits (infected but untreated) started to show the acute form of the disease (depression, reduced food intake, sneezing, conjunctivitis, respiratory distress, dyspnea, or even sudden death) with a mortality rate of 60%. However, rabbits treated with allicin (gp.3) and norfloxacin (gp.4) showed less severe clinical signs than did gp.2 with mortality rates of 40 and 30%, respectively. Rabbits treated with both allicin and norfloxacin (gp.5) were nearly healthy with much less severe clinical signs and a mortality rate of 10% till the time of scarification at the end of the experiment.

### 3.2. Erythrogram

At the end of experiment, there were significant decreases (*p* < 0.05) in RBC count, Hb concentration, PCV%, and MCHC values, while MCV values exhibited a significant increase (*p* < 0.05) in rabbits infected with *P. multocida* (gp.2), compared to controls (gp.1). However, rabbits of gps.3, 4, and 5 showed non-significant changes in the RBCs count, Hb concentration, PCV%, MCV, and MCHC% except Hb concentration and MCHC% showed significant increases in gp.5 compared to gp.2 (Table 1).

### 3.3. Leukogram

The total leukocytic, neutrophil, and monocyte counts showed significant increases (*p* < 0.05) with a significant decrease (*p* < 0.05) in the lymphocytic count and non- significant changes in eosinophil count in *P. multocida*-infected rabbits (gp.2), compared to controls (gp.1). The treated gps. (3, and 5) showed a non significant decrease (*p* < 0.05) in the previous parameters except the neutrophil count, which showed a significant decrease (*p* < 0.05) while total leukocyte count that showed a significant decrease (*p* < 0.05) in gp.5 compared to gp.2, while gp.4 showed a significant increases (*p* < 0.05) in the total leukocytic, neutrophil and eosinophil count with a non-significant changes in lymphocyte and monocyte counts compared to gp.2. The phagocytic activities (phagocytic% and phagocytic index) were significantly increased (*p* < 0.05) in *P. multocida*-infected rabbits (gp.2) compared to controls. The treated rabbits (gps.3, 4, and 5) exhibited significant increases in both parameters (*p* < 0.05), compared to the infected nontreated group; however, none of these treatments could restore the normal range concentrations as in the control group (gp.1) (Table 1).

### 3.4. Biochemical Analysis

Table 2 shows the detailed changes in different biochemical parameters in control, infected, and treated groups. Moreover, serum immunoglobulin (IgG and IgM) levels showed significant decreases (*p* < 0.05) in gp.2, while gps.3, 4, and 5 demonstrated significant increases (*p* < 0.05), compared to infected rabbits. The combination treatment restored the serum total protein concentration to the normal control level (as gp.1 rabbits).

### 3.5. Inflammatory Cytokines

Serum inflammatory cytokines (IL-6 and TNF-*α*) showed significant increases (*p* < 0.05) in gp.2 and significant decreases (*p* < 0.05) in the treated groups (4 and 5), except in the group receiving allicin (gp.3), which failed to improve serum TNF-*α* concentration, compared to infected nontreated rabbits (Table 2). The combination treatment could restore serum TNF-*α* concentration to normal control ranges (as gp.1 rabbits).

### 3.6. Hepatic Enzymes

The serum activities of ALT, ALP, and LDH significantly increased (*p* < 0.05) in the *P. multocida*-infected group compared to controls and significantly decreased (*p* < 0.05) in treated groups (3, 4, and 5) compared to gp.2. The total, direct, and indirect bilirubin concentrations showed significant increases (*p* < 0.05) in infected rabbits; however, it exhibited significant reductions (*p* < 0.05) in the treated groups (3, 4, and 5), compared to infected nontreated rabbits (Table 3).

### 3.7. Antioxidant/Oxidative Stress Markers

Group 2 rabbits showed significant reductions in serum GSH and SOD levels (*p* < 0.05) with a significant increase (*p* < 0.05) in MDA serum concentration, compared to controls, while the treated rabbits (gps.3, 4, and 5) exhibited significant increases (*p* < 0.05) in GSH and SOD concentrations with a significant decrease (*p* < 0.05) in MDA concentration, compared to infected nontreated rabbits (Table 4).

### 3.8. Histopathological Findings

**Figure 1:**
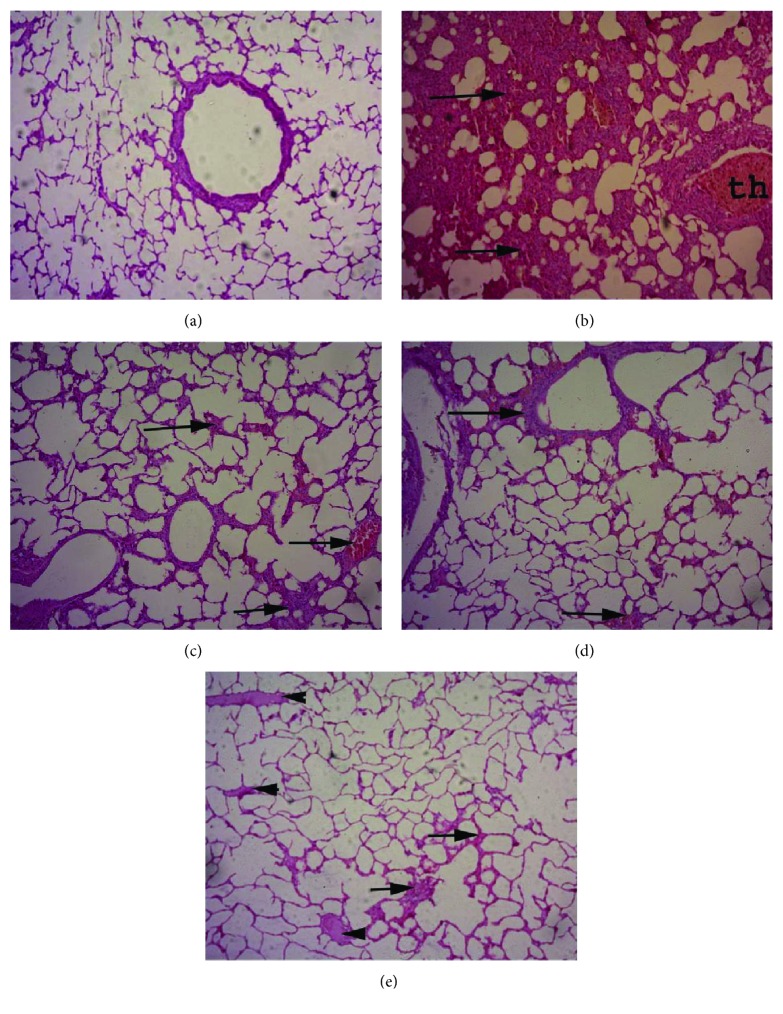
Shows lung sections from (a) normal control animals, (b) *Pasteurella*-infected group showing interstitial inflammatory reaction, (c) allicin-treated rabbits, (d) norfloxacin-treated rabbits, and (e) infected rabbits, treated with allicin-norfloxacin combination. Arrows refer to thickening of interstitial tissue with dilated capillaries and leucocytes, and arrowheads refer to edema. Hematoxylin and eosin stain; magnification: 100x.

The lung tissue of normal control rabbits exhibited normal bronchial and bronchiolar structures with normal alveolar and perialveolar capillaries. On the other hand, lung tissue sections from *Pasteurella*-infected rabbits showed severe peribronchitis, congested blood vessels, vascular thrombi, and vasculitis. Diffuse interstitial inflammatory reaction, alveolar collapse, tissue destruction, and compensatory emphysema were also observed. On the other hand, infected rabbits, treated with allicin, showed mild to moderate lesions as mild congestion and leukocytic infiltration of interalveolar capillaries with mild emphysema of alveoli and focal necrotic lesions. Rabbits that received norfloxacin after infection had mild thickening of the interalveolar septa with inflammatory cells and congestion. Moreover, group V rabbits (which received the combination treatment) showed mild edema between alveoli with scanty inflammatory cells and no necrosis or tissue destruction **(**Figure 1).

**Figure 2:**
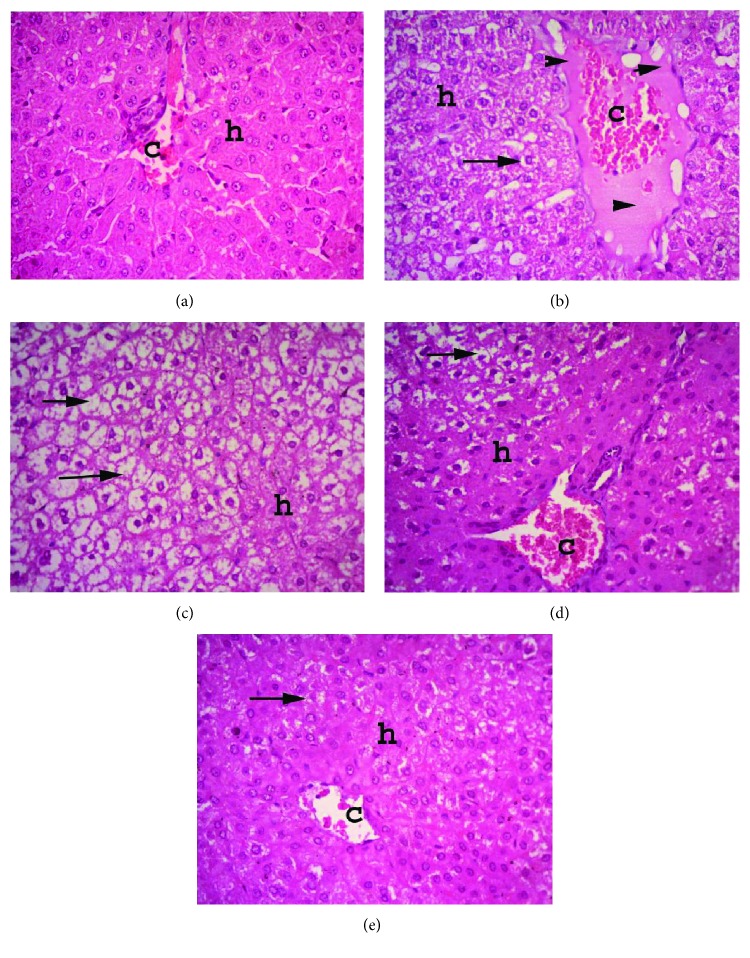
Shows liver sections from (a) normal control animals; (b) *Pasteurella*-infected group showing degeneration, necrosis of hepatocytes, and severe congestion of blood vessels; (c) allicin-treated rabbits; (d) norfloxacin-treated rabbits; and (e) infected rabbits, treated with allicin and norfloxacin combination. Arrows refer to thickening of interstitial tissue with dilated capillaries and leucocytes, and arrowheads refer to edema. Hematoxylin and eosin stain; magnification: 100x.

The liver tissue of normal control rabbits showed eosinophilic radiating hepatic cords around central veins. The hepatic cells had abundant cytoplasm and large centrally located nuclei. In contrast, liver sections from rabbits, infected with *Pasteurella*, demonstrated severe congestion of hepatic blood vessels along with perivascular edema, diffuse degeneration of hepatocytes, and focal necrosis. Infected rabbits, treated with allicin, showed diffuse vacuolar degeneration and mild to moderate congestion of blood vessels. Rabbits that received norfloxacin after infection had focal areas of degeneration with mild congestion of blood vessels. Further, sections from infected rabbits receiving the combination treatment showed fairly normal hepatic cords with focal-to-discrete hepatocytes suffering mild vacuolar degeneration **(**Figure 2).

## 4. Discussion

Rabbit husbandry requires good environmental conditions to reduce infection risks. Sneezing, nasal discharge, respiratory distress, and conjunctivitis were the common signs of *P. multocida* type B infection, observed in this study. Moreover, we detected frequent abscess formation in the lung tissue, bronchopneumonia, and septicemia, which may have been the main causes of morbidity and mortality in rabbits [31, 32]. Our results are in agreement with those of previous studies by Martino and Luzi [7], Palócz et al. [33], and Katoch et al. [15].

Allicin-treated rabbits exhibited less severe clinical signs and a lower mortality rate, probably related to the antibacterial and anti-inflammatory activities of allicin [34, 35]. Moreover, allicin was reported to scavenge free radicals and to inhibit the cysteine protease and thiol-containing protein in bacterial cells, inhibiting their growth [36, 37]. Similarly, infected rabbits treated with norfloxacin (100 mg/kg) showed marked reductions in the mortality rate and severity of clinical signs [38], probably due to the strong antibacterial activity of norfloxacin [39]. Interestingly, there was a marked reduction in the clinical signs and mortality rate in nearly all infected rabbits in the allicin-norfloxacin combination group, compared to either treatment alone.

Rabbits, infected with *P. multocida*, exhibited macrocytic hypochromic anemia (reticulocytosis), probably due to enhanced erythropoiesis as a response of the bone marrow to the increased blood loss in trachea-pulmonary hemorrhage, caused by septicemia [40]. Our results agree with those by Nassar et al. [41] who reported that there was a significant reduction in RBC count and PCV% in *P. multocida*-infected rabbits. Rabbits treated with a combination of allicin and norfloxacin showed an improvement in the picture of anemia, especially Hb level, which reflects reduction of the bacterial toxic effect on the bone marrow with decreasing or stopping the hemorrhage. The leukogram investigation revealed leukocytosis with heterophilia, and monocytosis in the *P. multocida*-infected group, which can be attributed to the body inflammatory response [40]; leukocytes were elevated in our study to overcome infection as they are the first line of the body's defense mechanism against any pathogenic agents.

Infections with *P. multocida* are usually associated with leukocytosis as a physiological response from the body to minimize the spread of infection [41–43]. Lymphopenia occurred in this study probably due to increased cytolysis produced by bacterial toxin and lymphocyte drainage into the infected tissues [44]. In contrast, rabbits treated with allicin alone showed a significant elevation in leukocyte count, reflecting the antibacterial and antitoxic effects of allicin [45]. Neutrophils showed a significant increase after allicin and/or norfloxacin treatment which may be a response to the increased serum concentrations of IL-6 and TNF-*α* [46–48].

The cellular immune response in the body increases physiologically in case of infection to destroy infective agents and minimize the spread of infection [42]. This study revealed a significant elevation in the phagocytic activity (phagocytic percent and phagocytic index) in *P. multocida*-infected rabbits. In agreement with El-Deeb and Elmoslemany [49], proinflammatory cytokines' serum levels (TNF-*α* and IL-6) were markedly elevated to enhance leukocyte migration into the infection site [46–48]. It has been reported that IL-6 increases in the blood after infection as an inflammatory response to regulate neutrophil and monocyte transition during the inflammation process [50, 51].

Allicin treatment significantly increased the phagocytic activity in infected rabbits, probably due to the ability of allicin to modulate the peripheral leukocytes' immune functions [52] through stimulating the proliferation of immune cells, lysozyme activities, and oxidative burst [53, 54], as well as enhancing the proinflammatory mediators like interferon-gamma and the expansion of CD4^+^ T cells [55]. Furthermore, allicin markedly alleviates the inflammation through reducing the production of TNF-*α* and IL-6 [56–58]. The antibacterial and anti-inflammatory effects of allicin may be explained by modulation of the cytokines and activating macrophages that controlled the infection.

Hypoproteinemia and hypoglobulinemia were observed in *P. multocida*-infected rabbits, which may be due to protein loss during hemorrhage. Anorexia and fever that result from the infection lead to increased protein catabolism and reduced protein synthesis by degenerated hepatocytes [33]. Allicin-treated groups showed significant improvements in serum protein and albumin levels, indicating the attenuation of hepatic injury and inflammation induced by *P. multocida* infection [35], hemorrhage reduction, and improvement of the animals' appetite.

Allicin-treated groups had an elevation in globulin, IgG, and IgM serum levels, reflecting the increased production of Igs from lymphoid organs to opsonize the *P. multocida* bacteria and limit the systemic infection [59]. It has been reported that allicin significantly enhances the immune response during infection through elevation of Ig levels [60]. Similarly, norfloxacin was an effective antibacterial agent, able to improve the protein, albumin, globulin, and Ig levels in infected rabbits.

Regarding enzyme activities and liver functions, ALT, ALP, LDH, and bilirubin showed significant elevations in *Pasteurella*-infected rabbits, possibly related to injury and degeneration of hepatocytes [61–63]. However, infected rabbits, treated with allicin and/or norfloxacin, showed marked reductions in serum ALT, ALP, LDH, and bilirubin levels, confirming the antibacterial activity of allicin and norfloxacin that reduced the damaging effects of bacteria on the liver [64].

Rabbits, infected with *P. multocida*, showed marked reductions in serum GSH and SOD with elevation of MDA levels, reflecting the increased lipid peroxidation and production of reactive oxygen species by bacterial infection [49]. Our results indicate that allicin treatment, alone or in combination with norfloxacin, ameliorated the oxidative stress and generation of free radicals in the infected rabbits represented by amelioration of lipid peroxidation and elevation of GSH and SOD levels through its antioxidative action [58, 65, 66]. Several garlic components, including allicin and selenium, have been shown able to attenuate the signaling pathways of reactive oxygen species and increase the endogenous antioxidant enzymatic activity [67–69]. Treatment with norfloxacin, alone or in combination with allicin, improved the antioxidant state and reduced the infection-induced oxidative stress.

## 5. Conclusion

The results of this study show that the clinically observed damage in rabbits infected with *P. multocida* can be ameliorated by allicin administration, probably through its antioxidant, anti-inflammatory, and immunostimulant effects. This protective effect could reduce the use of antibiotic in pets and livestock, reducing human exposure to antibiotic residues and bacterial resistance to antibiotics. Further, norfloxacin can be used for the effective treatment of pasteurellosis in rabbits. The combination of allicin and norfloxacin was more powerful in improving the *P. multocida*-induced alterations than each treatment alone.

## Figures and Tables

**Table 1:** The effect of allicin, norfloxacin, and their combination treatment in *Pasteurella multocida* type B infection in male white New Zealand rabbits on hematological parameters.

Parameters	Groups				
	Control	Infected	Infection + allicin	Infection + Nf	Infection + Nf + allicin
RBCs (10^6^/mm^3^)	4.51 ± 0.04^a^	3.63 ± 0.09^bc^	3.51 ± 0.03^bc^	3.43 ± 0.04^c^	3.58 ± 0.06^bc^
Hb (g/dl)	8.68 ± 0.05^a^	6.56 ± 0.7^c^	6.64 ± 0.12^c^	6.44 ± 0.07^c^	6.96 ± 0.08^b^
PCV%	36.40 ± 0.65^a^	31.98 ± 0.52^b^	31.56 ± 0.66^b^	30.78 ± 0.19^b^	31.20 ± 0.31^b^
MCV/FL	80.58 ± 0.76^b^	90.02 ± 1.18^a^	89.85 ± 2.21^a^	89.55 ± 0.79^a^	87.14 ± 1.65^a^
MCHC%	23.85 ± 0.36^a^	20.49 ± 024^c^	21.05 ± 0.26^c^	21.04 ± 0.19^c^	22.26 ± 0.17^b^
WBCs (10^3^/mm^3^)	6.35 ± 0.06^d^	7.35 ± 0.12^b^	7.15 ± 0.07^bc^	8.01 ± 0.26^a^	6.84 ± 0.06^c^
Neutrophil (10^3^/mm^3^)	1.31 ± 0.01^d^	3.41 ± 0.08^b^	3.00 ± 0.08^c^	3.82 ± 0.14^a^	2.87 ± 0.03^c^
Eosinophil (10^3^/mm^3^)	0.13 ± 00^c^	0.18 ± 0.03^bc^	0.17 ± 0.04^bc^	0.31 ± 0.02^a^	0.22 ± 0.04^b^
Lymphocyte (10^3^/mm^3^)	4.83 ± 0.06^a^	3.25 ± 0.13^bc^	3.53 ± 0.12^b^	3.42 ± 0.08^bc^	3.18 ± 0.03^c^
Monocyte (10^3^/mm^3^)	0.13 ± 00^b^	0.15 ± 0.07^a^	0.41 ± 0.03^a^	0.44 ± 0.07^a^	0.44 ± 0.03^a^
Phagocytic%	41.8 ± 0.49 ^e^	53.00 ± 0.32 ^d^	58.2 ± 0.200 ^c^	64.60 ± 0.400 ^b^	76.4 ± 0.51 ^a^
Phagocytic index	0.34 ± 0.01^e^	0.53 ± 0.01 ^d^	0.59 ± 0.003 ^c^	0.65 ± 0.013 ^b^	0.84 ± 0.005 ^a^

Values are represented as mean ± SEM (*n* = 10). Means within the same row carrying different superscripts (^a^, ^b^, ^c^, and ^d^) are significant at *p* < 0.05. Hb: hemoglobin; MCHC: mean corpuscle hemoglobin concentration; MCV: mean corpuscle volume; Nf: norfloxacin; PCV: packed cell volume; RBCs: red blood cells; WBCs: white blood cells.

**Table 2:** The effect of allicin, norfloxacin, and their combination treatment in *Pasteurella multocida* type B infection in male white New Zealand rabbits on some biochemical parameters.

Parameter	Groups				
	Control	Infected	Infection + allicin	Infection + NF	Infection + NF + allicin
Total protein (g/dl)	5.65 ± 0.06^a^	3.60 ± 0.07^c^	4.74 ± 0.08^b^	4.84 ± 0.04^b^	5.56 ± 0.02^a^
Albumin (g/dl)	3.99 ± 0.06^a^	2.50 ± 0.06^d^	3.33 ± 0.06^c^	3.28 ± 0.05 ^c^	3.58 ± 0.04^b^
Globulin (g/dl)	1.72 ± 0.40 ^b^	1.17 ± 0.01^e^	1.56 ± 0.03^c^	1.41 ± 0.03^d^	1.99 ± 0.03^a^
IgM (mg/dl)	26.00 ± 0.77^b^	13.80 ± 0.80^e^	19.60 ± 0.81^c^	16.6 ± 0.81^d^	33.80 ± 0.80^a^
IgG (mg/dl)	424.4 ± 1.47^b^	348.6 ± 1.17 ^e^	386.6 ± 2.48^c^	376.8 ± 2.33^d^	511.6 ± 3.25^a^
IL-6 (pg/ml)	224.80 ± 1.46^d^	325.00 ± 2.07^a^	250.00 ± 0.84^b^	243.60 ± 1.66^c^	246.00 ± 2.43^cb^
TNF-*α* (pg/ml)	91.20 ± 1.15^c^	120.60 ± 2.62^a^	117.80 ± 1.98^a^	105.20 ± 2.03^b^	92.40 ± 2.02^c^

Values are represented as mean ± SEM (*n* = 10). Means within the same row carrying different superscripts (^a^, ^b^, ^c^, ^d^, and ^e^) are significant at *p* < 0.05. Ig: immunoglobulin; IL: interleukin; NF: norfloxacin; TNF: tumor necrosis factor.

**Table 3:** The effect of allicin, norfloxacin, and their combination treatment in *Pasteurella multocida* type B infection in male white New Zealand rabbits on some hepatic markers.

Parameters	Groups				
	Control	Infected	Infection + allicin	Infection + NF	Infection + NF + allicin
ALT (U/L)	34.2 ± 1.06^d^	48.20 ± 0.20^a^	37.4 ± 0.87^c^	44.00 ± 0.44^b^	37.20 ± 1.07^c^
ALP (U/L)	66.25 ± 1.04^c^	124.41 ± 1.09^a^	83.74 ± 1.81^b^	68.19 ± 0.76^c^	68.22 ± 0.73^c^
LDH (U/L)	617.70 ± 4.03^d^	1105.63 ± 2.25^a^	800.56 ± 24.22^b^	683.74 ± 4.84^c^	646.44 ± 1.61^d^
Total bilirubin (mg/dl)	1.27 ± 0.004^d^	3.04 ± 0.004^a^	1.93 ± 0.024^b^	1.58 ± 0.124^c^	1.40 ± 0.044^d^
Direct bilirubin (mg/dl)	0.36 ± 0.014^d^	1.14 ± 0.029^a^	0.81 ± 0.013^b^	0.54 ± 0.040^c^	0.31 ± 0.002^d^
Indirect bilirubin (mg/dl)	0.91 ± 0.015^c^	1.90 ± 0.032^a^	1.12 ± 0.025^b^	1.04 ± 0.115^bc^	1.09 ± 0.043^bc^

Values are represented as mean ± SEM (*n* = 10). Means within the same row carrying different superscripts (^a^, ^b^, ^c^, and ^d^) are significant at *p* < 0.05. ALP: alkaline phosphatase; ALT: alanine transferase; LDH: lactate dehydrogenase; NF: norfloxacin.

**Table 4:** The effect of allicin, norfloxacin, and their combination treatment in *Pasteurella multocida* type B infection in male white New Zealand rabbits on oxidative stress markers.

Parameters	Groups				
	Control	Infected	Infection + allicin	Infection + NF	Infection + allicin + NF
GSH (mmol/L)	0.67 ± 0.02^a^	0.19 ± 0.01^d^	0.37 ± 0.03^c^	0.50 ± 0.03^b^	0.62 ± 0.02^a^
SOD (U/ml)	4.90 ± 0.15^a^	2.16 ± 0.09^c^	3.4 ± 0.17^b^	3.31 ± 0.14^b^	4.55 ± 0.09^a^
MDA (nmol/ml)	38.19 ± 0.44^d^	62.75 ± 1.32^a^	48.45 ± 0.83^b^	41.00 ± 1.08^c^	34.65 ± 0.68^e^

Values are represented as mean ± SEM (*n* = 10). Means within the same row carrying different superscripts (^a^, ^b^, ^c^, ^d^, and ^e^) are significant at *p* < 0.05. GSH: glutathione; MDA: malondialdehyde; NF: norfloxacin; SOD: superoxide dismutase

## Data Availability

The data used to support the findings of this study are available from the corresponding author upon request.

## Abbreviations

ALP: Alkaline phosphatase
ALT: Alanine transferase
GSH: Glutathione
Hb: Hemoglobin
IL: Interleukin
IN: Intranasal
LDH: Lactate dehydrogenase
MCHC: Mean corpuscle hemoglobin concentration
MCV: Mean corpuscle volume
MDA: Malondialdehyde
PCV: Packed cell volume
SOD: Superoxide dismutase
TNF: Tumor necrosis factor.
